# Background to the Intention to Photo-Post Food on Instagram: Potential Implications for Digital Food Marketing and Promotion of Healthy Eating

**DOI:** 10.3390/nu18213624

**Published:** 2026-10-31

**Authors:** Manuel Escobar-Farfán, Iván Veas-González, Jorge Bernal-Peralta, Elizabeth Emperatriz García-Salirrosas, Francisca Lasalas-Guerrero, Milenka Marín-Pastene

**Affiliations:** 1 Department of Administration, Faculty of Administration and Economics, Universidad de Santiago 3 de Chile (USACH), Santiago 9170020, Chile; manuel.escobar@usach.cl; 2 Departamento de Administración, Facultad de Economía y Administración, Universidad Católica 5 del Norte, Antofagasta 1270709, Chile; francisca.lasalas@alumnos.ucn.cl (F.L.-G.); milenka.marin@alumnos.ucn.cl (M.M.-P.); 3 Facultad de Administración y Economía, Universidad de Tarapacá, Arica 1000007, Chile; 4 Faculty of Management Science, Universidad Autónoma del Perú, Lima 15842, Peru; egarciasa@autonoma.edu.pe

**Keywords:** food photo-posting intention, Instagram food marketing, gastronomic experience, Stimulus–Organism–Response (SOR), consumer-generated content, self-expression, PLS-SEM

## Abstract

**Background/Objectives:** The rise in food content on social media changed how people find, assess, and select places to eat. While Instagram is known as one of the most important media for digital food advertising, little attention has been paid to the psychological and experiential factors that lead people to take and share pictures of food in restaurants. This paper examines the reasons for the intention to share food photos in restaurants on Instagram using the Stimulus–Organism–Response (SOR) and Uses and Gratifications Theory frameworks. **Methods:** Data for this study were collected using a cross-sectional quantitative design, and 243 Instagram users in Chile who had eaten in restaurants in the previous 6 months were surveyed. The model was analyzed using Partial Least Squares Structural Equation Modeling in SmartPLS 4, with aesthetic, functional, and symbolic design aspects as stimuli and self-expression, positive emotions, and satisfaction as organism states. **Results:** Of the eleven relationships examined, eight were supported. Gastronomic experience was the strongest factor influencing self-expression, clearly predicting both positive emotional arousal and satisfaction. Self-expression was the strongest direct predictor of the intention to post a picture, followed by satisfaction. Positive emotional arousal acted as a strong indirect predictor through satisfaction. The model explained 60.6% of the variance in the intention to post a picture. **Conclusions:** The research suggests that the intention to post food photos on Instagram is associated with the wish to express one’s self-identity and satisfaction with the food experience. Based on the findings, restaurant businesses can develop digital food marketing strategies centered on the dining experiences that customers want to share. Future research could examine whether these motives differ according to the nutritional characteristics of the food shared.

## 1. Introduction

Social media growth has transformed how food information is created, shared, and used. Among the numerous social media platforms, Instagram is considered a leader in food marketing, as it has over 2 billion monthly active users [1] and offers its audience the opportunity to create content rather than just consume it. The phenomenon of user-generated content (UGC) serves as a form of organic electronic word-of-mouth (e-WOM) through which restaurants can market themselves at little cost [2,3,4,5].

Food photo-posting is one of the most popular Instagram practices, in which users share pictures of their food and the places where they eat. As noted by Kemp [6], 62.3% of adult Instagram users use the platform to gather information about brands and products. In the food business, this is especially important because users look for photos posted by other consumers before choosing a restaurant [7,8]. The importance of this phenomenon is not limited to individual decision-making, as satisfied users share their experiences and create signals that influence the decisions of the entire online community [7,8,9].

Although this phenomenon has reached remarkable proportions, very little research has examined the actual motivations of consumers who post images of restaurant spaces on Instagram [3]. The bulk of research in food digital marketing focuses on the effects of published content on dietary behavior, whereas hardly any research addresses content production and the related psychological and experiential aspects of the phenomenon [10,11,12,13]. Knowing the reasons for this behavior has important practical implications for restaurant managers seeking to promote organic content generation, as well as broader implications for understanding the mechanisms by which food-related information spreads online.

This research fills this gap by establishing and validating a structural model explaining the causes of food photo-posting intention on Instagram. The research model of this study is based on the Stimulus–Organism–Response (SOR) theory [14,15], and the Uses and Gratifications Theory [16,17,18], and defines the restaurant’s design and the consumer’s culinary experience as environmental stimuli, self-expression, positive emotional arousal, and satisfaction, presented in terms of the organism’s condition, and the intent to post photos is seen as a behavioral reaction.

This research advances understanding by identifying mental and experiential factors that trigger the creation of food photo-posts and related e-WOM on Instagram. The findings can be useful not only for marketing purposes but also for nutrition research, a first step toward understanding the food information that circulates in virtual communities.

In addition to its marketing implications, this behavior is also relevant to the study of food communication. As user-generated food content increasingly complements official nutrition information, understanding the motivations behind its creation and sharing is a necessary starting point for future research on how food-related content circulates on social media. The present study does not assess the nutritional quality of the food shared. Rather, it examines the motivational processes that precede posting, which future studies could connect to the type of food depicted and to its effects on audiences.

## 2. Theoretical Background

### 2.1. Food Photo-Posting on Instagram as a Form of Digital Food Marketing

The intention to post pictures is the inclination of consumers to take and publish photos via Internet channels such as social media [7,8,19]. The reasons for this behavior include a mix of motives, such as social acceptance, sharing impressions, and a desire to belong to their social circle [7,16]. In the food service industry, posting pictures appears to be an opportunity for customers to show their preferences, culture, and identity [20,21], and at the same time serves as a facilitator of free self-marketing for the restaurant itself [3].

From a digital food marketing perspective, this behavior is unique in its impact and reach. Restaurant owners have long known that when customers post pictures of their food or ambiance on Instagram, they are doing free advertising and creating content that spreads beyond the restaurant’s official followers [3,4]. User-generated content is given so much credibility that it will never be matched by traditional advertising, and research has shown that people look for pictures taken by their peers before deciding where to eat [7,22].

The reasons why people post pictures of food do not appear to be the same for everyone. For some individuals, sharing unusual or attractive food pictures is simply a way to distinguish themselves from their surroundings and to show sophistication [23]. For other people, food photography is less about food itself than about the context in which food images are taken, an opportunity to maintain social ties and build a sense of belonging to a group with shared food tastes [23]. Despite the diversity of motivations for posting pictures of food, one idea holds true: posting pictures of food is a way to express oneself and influence how others perceive one’s personality [11,24,25]. From a marketing perspective, remember that this kind of posting is not accidental; it results from a person’s life experiences and their desire to express themselves and receive support from others. Thus, knowing what triggers that content-generation process is important, not only for restaurant marketing policies, but also for answering the wider questions on food-related information circulation on social media, what kinds of dining experiences get more attention, and how consumer-generated food content may relate to consumption norms in the digital world, a question that lies beyond the scope of this study but that our findings can help frame.

### 2.2. Theoretical Foundations of the Model

#### 2.2.1. The Stimulus–Organism–Response (SOR) Framework

The Stimulus–Organism–Response paradigm, proposed by Mehrabian and Russell [14], has been adopted as the theoretical basis for this research. The framework posits that environmental stimuli (S) elicit changes in the organism’s emotional and cognitive states (O), which in turn lead to approach or avoidance behavioral responses (R). Developed in environmental psychology to explain how environmental factors affect people’s behavior, SOR theory was later applied in consumer studies and hospitality management to examine the relationship between service-scape characteristics and customers’ perceptions [10,26,27]. In the context of restaurants, the physical environment, the venue’s aesthetic design, the functional organization of the space, and symbolic signs serve as environmental stimuli for customers.

These environmental variables influence the emergence of internal psychological states, such as emotions and evaluations, that ultimately determine whether a customer is likely to exhibit positive behavioral outcomes, such as recommending the venue, returning, or, as in our case, sharing photographs of the experience on social media [28,29]. The SOR model fits this study well because it highlights the mediating role of internal psychological states between the environment and behavior, which aligns with the multi-layered process by which restaurant design and gastronomic experience translate into photo-posting intention on Instagram.

#### 2.2.2. Uses and Gratifications Theory (U&G)

Uses and Gratifications Theory is a valuable tool for explaining why people post pictures on social media. This theory asserts that people use media to fulfill a variety of their needs, thus opposing the idea of treating customers as passive consumers [16,17,18]. While U&G has been studied in media research, it has proven especially relevant in social media studies, where researchers have analyzed the motivations behind people’s posting of pictures and content, e.g., self-presentation, entertainment, and socialization [18,30,31]. In particular, U&G is applied to food photography. Using this theory, researchers conclude that people post pictures of food on Instagram not just because they had a great experience and want to share it, but to fulfill a purpose. Thus, people post photos of food on Instagram to express their identity [3,32,33]. This behavior provides rewards such as self-expression, social acceptance, and a sense of belonging to a community of other food lovers, and these psychological conditions are central to this research, as they underlie the mediating role of the restaurant environment in the intention to post photos.

#### 2.2.3. Social Identity Theory

Social Identity Theory, initially proposed by Tajfel and Bruner [34] and further developed by Hogg et al. [35] holds that people derive a significant part of their identity from membership in specific social groups. The manner in which a group aspect of identity influences a consumer’s behavior is evident in their use of Instagram, where users share pictures of dishes, thereby signaling their membership in certain social reference groups, as well as their manner of living and culinary preferences [3,32,33]. Because posting dishes can convey one’s identity, it is seen as a source of information about one’s eating habits. Thus, when a consumer takes a photo of food on Instagram, they are not just sharing information about food but also communicating messages about their self-identity.

In terms of restaurant marketing, this suggests that restaurants that incorporate symbolic meaning into their decor and ambiance are better positioned to encourage customers to engage in self-expressive behavior, which results in the organic generation of content [33,36,37]. Regarding food information, it suggests that what is published about food and dining on Instagram is neither neutral nor representative of the food industry, but a selective representation driven by identity and shaped by creators’ symbolism and aesthetics.

### 2.3. Key Variables and Hypotheses

#### 2.3.1. Stimuli: Restaurant Design Dimensions and Gastronomic Experience

According to the SOR framework, stimuli are external factors that trigger consumer responses. In the restaurant context, the restaurant’s physical and symbolic attributes become the primary stimuli diners encounter. Referring to Homburg et al. [38] and Baek and Ok [39], restaurant design can be understood as comprising three dimensions, namely aesthetic, functional, and symbolic, that activate consumers’ responses in different ways. Along with these three design levels, the culinary experience can be seen as another factor that shapes the surroundings and encompasses everything the consumer senses.

Aesthetic design refers to how visually attractive a restaurant facility is to customers [11]. It encompasses all aspects of the space, such as lighting, colors, decorations, and overall aesthetics, that make a venue pleasant to the human eye [33,40]. Since it is difficult to assess a restaurant’s services before visiting, aspects such as visual appeal become more important, as people generally base their first impression of a restaurant on visual attractiveness [39,41]. When the visual signals are strong and coherent, they not only catch attention but also elicit emotional responses. Studies have shown that well-designed spaces lead customers to react positively to the venue, thereby sharing their impressions both before and after the visit, offline and online [42,43,44]. In the restaurant sector, design has become an important source of competitive advantage, as the ability to offer an aesthetically appealing experience has become a crucial part of its marketing strategy, especially in modern conditions, where client-generated visual content is used to attract newcomers [20,39,45].

Emotional processes following aesthetic design affect behavior. Research shows that the aesthetic design of a place can increase positive emotions in consumers, leading to heightened activation that drives certain types of behavior [10,11,39]. The first reaction is emotional rather than cognitive: people are excited without actually thinking about the image [10,33]. Also, when the aesthetics of a restaurant align with consumers’ tastes and understanding, another impulse, namely self-expression, is initiated [11,46,47]. Nevertheless, the question of whether aesthetic design propels self-expression in addition to eliciting certain emotions is insufficiently addressed in the literature [11], which motivates the following hypotheses:

**H1.**  *Aesthetic design has a direct and positive effect on consumer self-expression.*

**H2.**  *Aesthetic design has a direct and positive effect on positive emotional arousal.*

Functional design refers to the practical aspect of the restaurant environment—the extent to which the physical space conveys and fulfills the practical functions consumers expect [39,43]. It includes components such as table arrangements, traffic flow, sound comfort, and the overall ease of navigation and use of the environment [40]. Though functional characteristics might not be as immediately obvious and striking as aesthetic ones, they are nonetheless important in the justification and evaluation of consumption decisions; people use functional aspects as indicators of service quality and rational grounds for choosing one venue over another [39,48]. Most importantly, customers usually evaluate functional characteristics not through careful examination but through visual assessment; they know how a space functions from how it looks [39,49,50]. This indicates that functional design may not be aesthetically expressed. This reasoning supports the following hypothesis:

**H3.**  *Functional design has a direct and positive effect on positive emotional arousal.*

In the context of the restaurant environment, symbolic design is considered the dimension of design laden with meaning. This is because while aesthetic design refers to beauty and functional design concerns the utility of the restaurant environment, symbolic design concerns the extent to which the physical environment conveys a certain lifestyle, set of values, or social identity with which individuals feel identified [33]. By investing in the domain of symbolic design, restaurants do not merely create appealing/pleasing spaces but rather build environments that reflect the identities of different consumers by signaling, “This is the type of space where one like you would go” [38,51]. Upon finding such correspondence, consumers experience a strong sense of recognition through alignment between their self-concept and the symbolic meaning of the restaurant environment [33,37].

It is because of this very mechanism that the relationship between symbolic design and the actual posting of photos is so strong, the restaurant, which has a design that echoes the lifestyle that conforms to the identity of the consumer, offers what has recently been called an Instagrammable setting, as it provides a kind of symbolic cues, which resonate with the self-image of the consumer so accurately that taking photos at the venue becomes almost second nature [33]. The emotional engagement that results from that symbolic fit links the design to the sharing instinct and, thus, makes symbolic design an important predictor of online content creation. This leads to our following hypothesis:

**H4.**  *Symbolic design has a direct and positive effect on positive emotional arousal.*

Gastronomic experience is a vast and multifaceted concept, encompassing everything a customer thinks, feels, and evaluates when dining in a restaurant [52]. Researchers have recognized three different dimensions of the gastronomic experience: presentation dimension, including food appearance, color, texture, and plating; situational dimension, which involves the ambiance, spatial arrangement, location, and interactivity with the staff; and individual dimension, where personal perception, feelings, and time spent by a customer while dining are taken into account [53,54]. It can be concluded that all these dimensions indicate that the gastronomic experience is a unique and subjective phenomenon; it can never be identical for different customers in a restaurant [55].

The gastronomic experience matters to this research because it can shape multiple consumer responses simultaneously. Past studies have concluded that positive gastronomic experiences lead to consumer loyalty, satisfaction, positive e-WOM feedback, and broader behavioral intentions [9,56,57]. When analyzed through the SOR framework, the gastronomic experience emerges as a unique stimulus because, unlike the design elements discussed earlier, it affects the consumer as a whole [27]. In particular, the rich gastronomic experience facilitates self-expression by strongly incentivizing consumers to share it with others: an experience that reflects their tastes, positions them as representatives of a certain lifestyle, and affirms their identity as sophisticated patrons of great dining [20,23,58]. Interestingly, gastronomic experiences that meet or exceed consumers’ expectations lead not only to positive emotional arousal, the degree of excitement and hedonistic pleasure that makes consumers eager to share online [29,59,60], but also to satisfaction, which is the psychological state achieved when actual performance meets consumers’ expectations [61,62]. The outlined multifaceted impact of gastronomic experience leads to three specific hypotheses.

**H5.**  *Gastronomic experience has a direct and positive effect on self-expression.*

**H6.**  *Gastronomic experience has a direct and positive effect on positive emotional arousal.*

**H7.**  *Gastronomic experience has a direct and positive effect on satisfaction.*

#### 2.3.2. Organism States: Self-Expression, Positive Emotional Arousal, and Satisfaction

In the SOR framework, organism states denote the internal psychological states of organisms, cognitive, emotional, and evaluative, that are used to process environmental stimuli and convert them into behavior. The current study uses three organismic states to mediate the link between the restaurant environment and the intention to post photos: self-expression, positive emotions, and satisfaction. Each state reflects a different aspect of consumers’ internal psychological state, but together they form an integrated psychological system that drives motivation to create and share visual content on Instagram [18,33].

Positive emotional arousal refers to a state of heightened activation, characterized by excitement, enthusiasm, and hedonic pleasure, that customers experience when a restaurant exceeds their expectations for the service they receive [39,63]. From a psychological reasoning perspective, emotions guide cognitive processing: the more positive the emotions, the better future events are interpreted [39,64]. In the restaurant context, the physical setting is a major factor in emotional activation: the exciting, meaning-making space creates sensory pleasure and excitement that go beyond mere rational evaluation of service [10,65]. This emotional activation also shapes how satisfied diners feel with the service [63]. In this way, the restaurant’s emotional atmosphere informs the evaluation of the experience itself, which leads to the following hypothesis:

**H8.**  *Positive emotional arousal has a direct and positive effect on satisfaction.*

Self-expression holds that consumers naturally want to express their preferences, style, and values through their behavior and consumption choices to present themselves as unique individuals [3,47]. More specifically, when they visit a certain restaurant, the very choice of it is considered by patrons to serve a function beyond the utilitarian—it shows who the consumers are and how they would like to be perceived [66,67]. Their likelihood of recommending a restaurant increases significantly when they feel that “this place is made for them” and want to share their experience with their networks [3,68]. Social networks, especially Instagram, greatly intensify this process. It means that pub-lovers do not just document their visit but perform their identity in front of their audience, seeking recognition from them [33,47]. Studies have shown that self-expression is one of the most important motives behind the intention to take pictures and post them on Instagram among different food industry actors [11,20,33]. This supports the following hypothesis:

**H9.**  *Self-expression has a direct and positive effect on photo-posting intention on Instagram.*

Positive emotional arousal is also essential for content generation, as research shows that emotionally intense experiences are more likely to be shared; they attract attention and spread more quickly in social networks [33]. The emotional climate of the restaurant may therefore act not only as an antecedent of satisfaction but also as a direct source of sharing behavior, which leads to the following hypothesis:

**H10.**  *Positive emotional arousal has a direct and positive effect on photo-posting intention on Instagram.*

Satisfaction is the ultimate evaluation of the food experience, a psychological state that occurs when consumers believe the restaurant’s actual performance meets or exceeds their expectations [61,69]. Satisfaction is regarded as one of the most reliable predictors of post-consumption behavioral intentions in the service context, including intentions to recommend, return, or publicly endorse the restaurant [70,71]. For restaurants, several experiential components shape satisfaction, including menu quality, service quality, and ambiance [72]. A satisfied customer is not necessarily loyal, but they can be an influential communicator. The link between satisfaction and e-WOM generation is widely acknowledged: a satisfied customer will spread the word about their dining experience through verbal communication or social networks [73,74]. For instance, someone sharing their gastronomic experience on Instagram may also want to create and share images that reflect that experience, thereby receiving positive reinforcement from the restaurant, as these images help the restaurant promote itself organically. This reasoning supports the final hypothesis of the model:

**H11.**  *Consumer satisfaction has a direct and positive effect on photo-posting intention on Instagram.*

Figure 1 illustrates the theoretical framework for this research. The model classifies four stimulus factors (aesthetic design, functional design, symbolic design, and gastronomic experience) on the left side of the figure. The three organism states (self-expression, positive emotional arousal, and satisfaction) emerge from the stimulus factors in the center of the figure, thereby leading to photo-posting intention on Instagram as a behavioral response depicted on the right. The arrows represent the eleven hypothesized relationships (H1–H11), indicating the expected positive links among different conceptions. Overall, the model describes the psychological process by which relationships between restaurant and dining experiences lead consumers to create visual food content on Instagram.

## 3. Materials and Methods

### 3.1. Sample

The researchers used a quantitative cross-sectional design to analyze the antecedents of the intention to post food pictures on Instagram in the restaurant context. The target population was Instagram users aged 18 or older who had visited a restaurant in Chile within the 6 months before data collection. This criterion ensured that participants’ responses came from recent, firsthand gastronomic experience. Researchers used non-probability convenience sampling and invited participants to complete an online survey created in Google Forms and shared on social media platforms, primarily Instagram, WhatsApp, Facebook, and TikTok, from October to November 2023.

Eligibility was verified using screening questions at the beginning of the questionnaire regarding Instagram usage and restaurant visits over the previous six months; respondents who did not meet these criteria did not proceed to the construct items. Prior experience posting photos of food or restaurants was not required, given that the dependent variable is the intention to post. Respondents were asked to think of the restaurant they liked best or that had most captured their attention among those visited in the last six months, and to answer all construct items with that restaurant in mind. Of the 251 responses received, eight were excluded because the respondents did not finish the survey, leaving 243 valid responses.

The minimum sample size was estimated in G*Power version 3.1 using an F test for multiple linear regression (f^2^ = 0.10, α = 0.05, statistical power = 0.95) with seven predictors. This is a conservative setting, as the largest number of predictors pointing at any endogenous construct in the model is four. The minimum required sample was 226 cases, which the final sample of 243 exceeds. Ethical concerns were addressed through informed digital consent obtained at the start of the survey. Participation by respondents was voluntary and anonymous; no personal data were collected during the research.

### 3.2. Measurement Instrument

The questionnaire used in the study consisted of 31 indicators measuring eight latent constructs, namely the following: aesthetic design; functional design; symbolic design; gastronomical experience; self-expression; positive emotional arousal; satisfaction; and intention to post photos. All the items were adapted from other reliable sources in the hospitality and consumer behavior literature and are summarized in Table 1 of the study. Respondents rated each item on seven-point Likert scale ranging from 1 (strongly disagree) to 7 (strongly agree). The items were translated and culturally adapted using a forward–backward translation procedure. First, the research team translated the original English items into Spanish and adapted their wording to the Chilean restaurant context, referring each statement to “this restaurant”, meaning the establishment chosen by each respondent. Because the items were drawn from different studies, the content validity of the adapted questionnaire was then assessed through academic expert judgment [75]. A panel of five researchers with doctoral degrees and experience in marketing and consumer behavior reviewed the 31 items and qualitatively assessed their clarity, their suitability for the restaurant context, and their conceptual equivalence with the original English versions. Their observations led to minor wording adjustments in some items without changing the items’ content. Next, the research team, together with a bilingual English/Spanish translator, back-translated the Spanish items into English and compared this version with the original items to identify discrepancies in meaning. Finally, the questionnaire was pretested with 30 Instagram users who met the eligibility criteria, who assessed how well they understood each indicator. Based on their feedback, minor wording changes were made to improve comprehension before the final questionnaire was administered. Pretest participants were not included in the final sample. The complete wording of the items in the original English version and in the Spanish version administered to respondents, together with their sources, is provided in Supplementary Table S1.

### 3.3. Statistical Analysis

Partial Least Squares Structural Equation Modeling (PLS-SEM) was used to analyze the data using SmartPLS 4 software [77]. There were two main reasons for the preference for PLS over covariance-based SEM: first of all, the research model was exploratory, since it was trying to find what the predictors of a behavior with little previous research are, and secondly, the data distribution was not characterized by multivariate normality, which PLS can easily deal with [78].

According to the two-step process recommended by Hair et al. [78], the investigation was conducted in two stages. First, the measurement model underwent reliability and validity testing. Internal consistency reliability was assessed using Cronbach’s alpha (α > 0.70) and composite reliability (CR > 0.70). Convergent validity was verified through the average variance extracted (AVE > 0.50), and the Fornell–Larcker criterion and Heterotrait–Monotrait ratio (HTMT < 0.85) tests served for the evaluation of discriminant validity [79]. Assessment of model fit was conducted using the Standardized Root Mean Square Residual (SRMR) [80].

During the second phase, the structural model was evaluated for its path coefficients (β) and statistical significance through bootstrapping 5000 subsamples of data at a 5 percent significance level. In addition, the coefficient of determination (R^2^) was used to assess the model’s explanatory power, following guidelines from Hair et al. [78]: 0.25 indicates weak explanatory power, 0.50 suggests moderate explanatory power, and 0.75 indicates strong explanatory power.

## 4. Results

### 4.1. Sample Profile

Table 2 presents the sample’s sociodemographic characteristics. Most participants were women (56.4%), 42.4% identified as men, 0.8% were non-binary, and 0.4% chose not to identify their gender. In terms of income, the largest share (25.1%) of respondents reported a monthly income between CLP 880,001 and CLP 1,320,000, while the second-largest share (21.0%) reported earning no more than CLP 440,000. Most participants lived in the city of Antofagasta, Chile. Additionally, respondents selected more than 40 establishments. The most frequently selected restaurant accounted for 11.1% of responses, and close to 30 establishments were mentioned only once. Restaurant format, price range, and service level were not recorded, as noted in the Limitations subsection.

### 4.2. Measurement Model: Reliability and Convergent Validity

Table 3 presents the measurement model evaluation results, including outer loadings, Cronbach’s alpha, composite reliability, and average variance extracted (AVE) for all eight constructs. All indicator outer loadings exceeded the minimum criterion of 0.707 [78,81], ranging from 0.813 to 0.990 across all items used in the study (31 items). This indicates that all the indicators are positively and strongly correlated with their respective latent variable. Table 3 presents the full factor model output.

Regarding internal consistency, the Cronbach alpha values for all constructs ranged from 0.902 to 0.989, well above the recommended minimum threshold of 0.70 [78,81]. Similarly, composite reliability values (rho_a and rho_c) ranged from 0.911 to 0.992, again exceeding the 0.70 benchmark. Cronbach’s α exceeded 0.95 for five constructs (α = 0.960 to 0.989). Because such values may indicate indicator redundancy [82], we examined item wording, inter-item correlations, and item-level diagnostics (Supplementary Tables S2 and S3). The clearest overlap was in satisfaction, where the three items all refer to the decision to visit the restaurant (r = 0.962 to 0.970). A sensitivity analysis that removed redundant items changed path coefficients by no more than 0.024 and did not alter any hypothesis test (Supplementary Table S4), which confirms the stability of the results. All items were adapted from previously published scales (Supplementary Table S1); however, this overlap indicates that some constructs were measured in a narrow form, which the study acknowledges as a limitation.

To validate convergent validity, AVE was used to determine the percentage of variance explained by a construct relative to its measurement error [83]. The AVE values in the study range from 0.772 to 0.978, well above the 0.50 threshold. This implies that each construct explains more than half of its indicators. Overall, Table 3 shows that the measurement model met the requirements for standard reliability and convergent validity tests.

### 4.3. Discriminant Validity

Discriminant validity was evaluated through a combination of methods. First, the Fornell–Larcker criterion necessitates that the square roots of AVE values of each construct should surpass correlations with other constructs in the model criterion [83]. As shown in Table 4, the diagonal elements (square roots of AVEs) exceeded the off-diagonal correlations, supporting discriminant validity for every construct pair.

Second, HTMT has been applied in accordance with the recommendations of Henseler et al. [79], who recommended a cautious cutoff of 0.85 for models comprising conceptually diverse constructs. As highlighted in Table 5, all HTMT values are below this level, ranging from 0.324 to 0.817. The maximum HTMT value (0.817) is for gastronomical experience and satisfaction, confirming the theoretical closeness of these constructs while remaining within acceptable limits. Table 4 and Table 5 provide sufficient evidence of discriminant validity, as all eight constructs measure distinct theoretical aspects.

### 4.4. Model Fit

To assess model fit, we used the Standardized Root Mean Square Residual (SRMR). The values for the saturated and estimated models, shown in Table 6, were 0.040 and 0.072, respectively, both below 0.08 [80]. The Normed Fit Index (NFI) was also acceptable: the saturated model was 0.877, and the estimated model was 0.873. The results confirm that the proposed model closely matches the data structure.

### 4.5. Structural Model and Hypothesis Testing

The coefficient of determination (R^2^) assesses the model’s explanatory power. As shown in Table 7, satisfaction had the highest explained variance (R^2^ = 0.711), indicating that the model has strong explanatory power, according to Hair et al. [78].

Photo-posting intention on Instagram (R^2^ = 0.606) and positive emotional arousal (R^2^ = 0.600) also had moderate-to-high explanatory power. Self-expression had a lower explained variance (R^2^ = 0.177), indicating that the stimuli in the model explain only a small share of this organism state.

Table 8 shows the structural model, with path coefficients obtained using SmartPLS 4. The bootstrap method, utilizing 5000 sub-samples at a significance level of *p* < 0.05, was used to assess the structural relationships. The overall outcomes regarding hypothesis testing are provided in Table 8. Of the eleven hypotheses, eight were supported. The gastronomic experience was shown to be the most significant predictor regarding the paths of stimuli leading to organism response, exerting a strong positive influence on self-expression (H5: β = 0.367, t = 4.982, *p* < 0.001), positive emotional arousal (H6: β = 0.567, t = 8.862, *p* < 0.001), and satisfaction (H7: β = 0.501, t = 5.844, *p* < 0.001), thus confirming its importance as the major stimulus in the restaurant context. Among the design parameter dimensions, the aesthetic design affected positive emotional arousal considerably (H2: β = 0.174, t = 2.941, *p* = 0.003), while the symbolic design appeared to have a significant influence on positive emotional arousal (H4: β = 0.124, t = 2.142, *p* = 0.032). In comparison, the aesthetic design did not make a statistically significant prediction of self-expression (H1: β = 0.078, t = 0.963, *p* = 0.335), and the functional design did not show any significant influence on positive emotional arousal (H3: β = 0.018, t = 0.276, *p* = 0.782), which led to the rejection of both.

Among the organism-to-organism relationships, positive emotions were a major predictor of satisfaction (H8: β = 0.400, t = 4.498, *p* < 0.001). In the organism-to-response connection, self-expression was the strongest predictor of the intention to post a photo (H9: β = 0.556, t = 10.580, *p* < 0.001); satisfaction also positively predicted this intention (H11: β = 0.257, t = 3.319, *p* = 0.001). At the same time, positive emotions did not directly affect the intention to post a photo (H10: β = 0.104, t = 1.210, *p* = 0.226). Indeed, positive emotions indirectly trigger the behavioral response through satisfaction rather than serving as a direct trigger.

## 5. Discussion

The main aim of this research was to identify and confirm the factors affecting the intention to post food photos on Instagram in the restaurant context, using a model grounded in the SOR framework and Uses and Gratifications Theory. The findings of this research show how restaurant context and dining experience relate to the willingness to create and upload visual content on social media. Of the eleven proposed hypotheses, eight were confirmed, and the model accounted for 60.6% of the variance in the intention to post photos, indicating both the strength of the identified factors and the complexity of the phenomenon, which is associated with various motivations.

The most notable result in the model is the dominant role of gastronomic experience as the stimulus with the widest impact on the respondents’ internal states. Gastronomic experience showed high predictive power for the respondents’ self-expression (β = 0.367), positive emotional arousal (β = 0.567), and satisfaction (β = 0.501), thereby confirming all three hypotheses (H5–H7). Such results align with earlier findings indicating that gastronomic experience serves as a multidimensional stimulus with a unique impact, shaping the consumer at cognitive, affective, and social levels throughout the dining process [27,56]. The main contribution of this research is the identification of the precise mechanisms by which gastronomy serves as a trigger for posting pictures on food-related social media. Gastronomy operates through three simultaneous cognitive influences: the need for identity formation, the experience of positive affect, and the fulfillment of one’s desires. The interaction among these mechanisms clarifies why gastronomy has become such a powerful motive for the generation of food content in social media—if the food is tasty, enjoyable, and important for a user, it will be so difficult to resist the urge to share it.

Self-expression is the strongest direct predictor of the intention to post photos (β = 0.556, *p* < 0.001), confirming H9, which has the highest path coefficient in the model. This finding confirms the theoretical role of self-expression in Use and Gratification Theory [16] and past empirical studies on the restaurant industry [11,20,33]. It illustrates one of the well-established patterns in the social media literature, which shows that consumers use Instagram not only to document experiences but also to present themselves and express their beliefs, tastes, and social relations to their online community [3,68]. Thus, the restaurant experience becomes the subject of the story, the main material for identity construction, with the photograph serving as the means of its telling. The main conclusion from this reading is that people become content creators not because of the experience itself, but because they want the experience to be seen as reflecting who they are and their identity.

Satisfaction also emerged as a significant direct predictor of photo-posting intention (β = 0.257, *p* = 0.001), thus confirming H11 and providing further evidence of the established connection between post-consumption satisfaction and positive intentions in the services-related literature [73,74]. More importantly, satisfaction was an important mediator in the model: positive emotions directly predicted satisfaction (β = 0.400, *p* < 0.001), which in turn predicted photo-posting intention. This mediating relationship clarifies why H10, which claimed that positive emotions would predict photo-posting intention, must be rejected.

Previous studies have shown that positive emotions promote sharing behavior online [33]. This study shows that when sharing photos of food at restaurants, emotional excitement alone is not enough. It must be determined whether this excitement results in a feeling of satisfaction, in other words, whether the individual feels that their experience met their expectations, which in turn leads to the desired action. This distinction is both theoretically and practically important because it implies that the overall quality of the experience motivates sharing.

Among the design dimensions, aesthetic design (H2: β = 0.174, *p* = 0.003) and symbolic design (H4: β = 0.124, *p* = 0.032) were shown to be able to significantly predict positive emotional arousal, but functional design was not (H3: β = 0.018, *p* = 0.782). Rejection of H3 is in line with the theoretical distinction between affective and utilitarian responses to design, according to which functional characteristics address practical needs and reduce uncertainty but fail to trigger the hedonic excitement typical of positive emotional arousal [39,64]. In cognitive evaluation, functional design is perceived rationally because it informs consumers about a restaurant’s efficiency rather than eliciting their feelings about the dining experience. On the contrary, aesthetic and symbolic designs have an affective nature, as their images elicit immediate pre-cognitive emotional responses stemming from the hedonic nature of social sharing on social media [10,33]. The rejection of H1, which states that aesthetic design directly influences self-expression, should also be mentioned, as it shows that aesthetic-induced emotional arousal, rather than direct influence on identity expression, mediates the relationship between aesthetic design and content generation. Simply put, an aesthetically pleasing restaurant does not provide an experience of self-expression but instead excites consumers; that excitement leads to sharing after they complete their culinary experience.

Attributes of restaurant design- mainly aesthetic and symbolic dimensions cause positive emotional arousal that stimulates the satisfaction process. The gastronomic experience creates a reality in which self-expression, positive emotional arousal, and satisfaction emerge. At the moment of decision-making about whether to share their experience, the most important factors influencing consumers are self-expression and satisfaction.

### 5.1. Theoretical and Practical Implications

From a theoretical perspective, this research paper has made several contributions at the intersection of digital food marketing, consumer behavior, and social media research. First and foremost, this research presents a comprehensive SOR model of food photo-posting intention that goes beyond previous work restricted to single predictors or dyads. By providing an understanding of all stages of the process, ranging from restaurant atmosphere to content generation, across various organism states, the proposed model yields a more complex and deeper behavioral explanation for consumers’ posting of food pictures on Instagram than was previously available in the literature. The explanatory power of the model (R^2^ = 0.606 for food posting intention) compares favorably with results from studies in the hospitality and social media domain [11,33], indicating that the SOR framework and U&G theory provide an effective and applicable theoretical basis for the chosen topic. These contributions refer to the formation of posting intention. Intentions do not always translate into action, and situational factors at the table, such as the company, time pressure or privacy concerns, may widen the gap between the two. The model should therefore be read as an explanation of the motivational stage that precedes posting rather than of posting itself.

Second, the study advances the ongoing debate on consumer-generated digital food marketing by highlighting food photo-posting not as an insignificant social media activity, but as a significant means of organic food communication that shapes how gastronomic engagements frame dining experiences and how different food categories are perceived within the network. According to the results, the content produced and shared by consumers is influenced by different identity-affiliated factors along with quality of experiential or subjective enjoyment: not all experiences connected with restaurants end in a social media post, with only those that successfully fulfill the requirements of format suitability, identity match, and positive culinary impression doing so. Amplifying consumers’ posts has implications beyond marketing, as it raises questions about the culinary environment’s image that emerges and spreads through the forms consumers create on Instagram, and how this image influences social media users’ eating habits and aspirations within the community.

At the policy level, these results may also be of interest to social marketing campaigns and public health communicators who use digital channels, although any application to healthy eating requires further research. Since the strongest predictors of posting intention were self-expression and satisfaction rather than aesthetic appeal, future studies could test whether healthy eating messages that connect food choices with identity and with satisfying dining experiences are more likely to be shared than messages based only on visually attractive images. Because the present study did not assess the nutritional quality of the food depicted, food choice or dietary intake, these possibilities should be understood as hypotheses for future work rather than as recommendations derived from our data.

Thirdly, the research finding that positive emotional elevation affects photo-sharing only indirectly, through factors such as subjective satisfaction, offers new insights into the emotions underlying sharing decisions on social media in service industries. This finding implies that when posting restaurant experiences on Instagram, consumers think critically and do not act on impulse; they share food experiences only after assessing the value of a particular gastronomic event. From the perspective of viral content theory, this finding indicates that when consumers share posts featuring food photos, emotions serve as a motive only during the evaluation process, not as immediate trigger for posting.

In practical terms, restaurant managers and food service marketers should pay particular attention to the dominant role of gastronomic experience, the stimulus with the strongest influence on the three organism states. Thus, the analysis concludes that investment in the dining experience should be prioritized over other marketing mechanisms. A good restaurant that manages to surprise its customers gives them many reasons to share their experience: they want to tell something about themselves, feel emotionally connected, and feel satisfied. While good design can attract customers’ attention on Instagram, it cannot replace a dining experience that truly delivers.

However, the presence of aesthetic and symbolic cues in the design is significant. The significant influence of symbolic design on positive emotional arousal proves the necessity to create spaces that would be personally meaningful and resonate with the identity of the target consumer, the so-called “Instagrammable” design in the recent literature [33]. Restaurant managers need to know not only what their restaurant’s design is like but also what it signifies about the people eating there. The design of the venue, which communicates the values, aspirations, and lifestyles of the target segment, is more likely to motivate consumers to express themselves and, consequently, share on social media.

Lastly, the findings suggest that understanding the motives behind consumer-generated food content is relevant for digital food marketing and may also inform future public health research. If content on Instagram about food experiences is linked to identity-related and satisfaction-related motives, some restaurants or eateries may become more visible than others. Researchers and health practitioners interested in how digital platforms shape nutritional perceptions could therefore examine both the content displayed on Instagram and the reasons behind its creation.

### 5.2. Limitations and Future Research Directions

The limitations of this study must be considered when interpreting its conclusions. First of all, the sample was selected non-randomly and is heavily focused on the city of Antofagasta (Chile). While this method has merits in exploratory research design, it limits the applicability of the results to other geographical, cultural, and socioeconomic contexts. Future researchers should try to replicate the findings using larger samples that represent geographic diversity, including comparisons between countries with different food identities and social media practices. Thus, researchers may need to study larger groups of respondents from diverse backgrounds.

Second, the study uses intention to post as the dependent variable rather than actual posting behavior. Although intentions are often used as a substitute for behavior in consumer studies, the transition from intention to action is not direct and can be affected by factors such as privacy concerns, knowledge of the platform’s algorithms, or specific circumstances that may or may not allow the intention to be realized. Thus, further research should measure the actual posting behavior (for instance, using the digital trace method or the longitudinal method).

Third, the model ignores generational or demographic differences among Instagram users. It is essential to determine whether the associations described in the current research also hold for older consumer segments. In particular, it would be interesting to see whether self-expression is also the leading variable among younger users, who are considered the most active on Instagram.

Fourth, the cross-sectional design does not allow causal inferences, so the relationships reported here should be interpreted as associations consistent with the proposed model. Fifth, respondents evaluated different restaurants, and we did not record restaurant format, price level, or service style, so this heterogeneity could not be controlled for. Future studies could compare restaurant types or include them as moderators. Sixth, the very high internal consistency of some constructs reflects semantic overlap among their items. Satisfaction captured satisfaction with the decision to visit the restaurant rather than the full evaluation of the dining experience, functional design captured only the perceived proper functioning of the design, and the gastronomic experience scale was dominated by items on sensory food quality. Although a sensitivity analysis showed that this redundancy did not alter the results, future studies should use shorter or more differentiated scales that capture the full conceptual breadth of these constructs.

Finally, this study did not distinguish between healthy and unhealthy food content in the photographs respondents intended to post, nor did it assess food choice, dietary intake, or audience responses to the posted content. In future studies exploring the use of digital methods for the promotion of healthy eating, it would be interesting to find out whether the identified psychological motives (self-expression and satisfaction) have a different impact when the shared content consists of healthier food exclusively, and whether restaurants and public health campaigns can design gastronomic experiences and symbolic cues in a manner that would make healthy eating options more “shareable.”

## 6. Conclusions

This research aimed to understand the motives behind Instagram users’ decisions to document their dining experiences, a question that connects three critical fields: consumer psychology, hospitality management, and digital food marketing. Using an SOR-based PLS-SEM technique validated with 243 Chilean Instagram users, the study has shown that the intention to photograph and post food is associated with a structured set of motives related to self-identification and experience quality.

Gastronomic experience was the most significant factor in this process, as it stimulated self-identification, emotional excitement, positive feelings, and satisfaction for the diner. Self-identification was the primary direct predictor of posting intention, indicating that the intention to share food photographs is closely tied to identity expression. Satisfaction served as an outcome of the restaurant visit and an antecedent of the intention to share photos, while emotional excitement acted indirectly, meaning that feelings generated by a restaurant encounter influence the intention to share online.

Among restaurant design characteristics, aesthetic and symbolic elements, but not functional ones, were positively associated with emotional excitement, supporting the need to create places that resonate with consumers on both visual and semantic levels. Importantly, the research raises broader questions about how digital food information spreads. With digital platforms, users can produce content by focusing on the intent behind sharing food content.

## Figures and Tables

**Figure 1:**
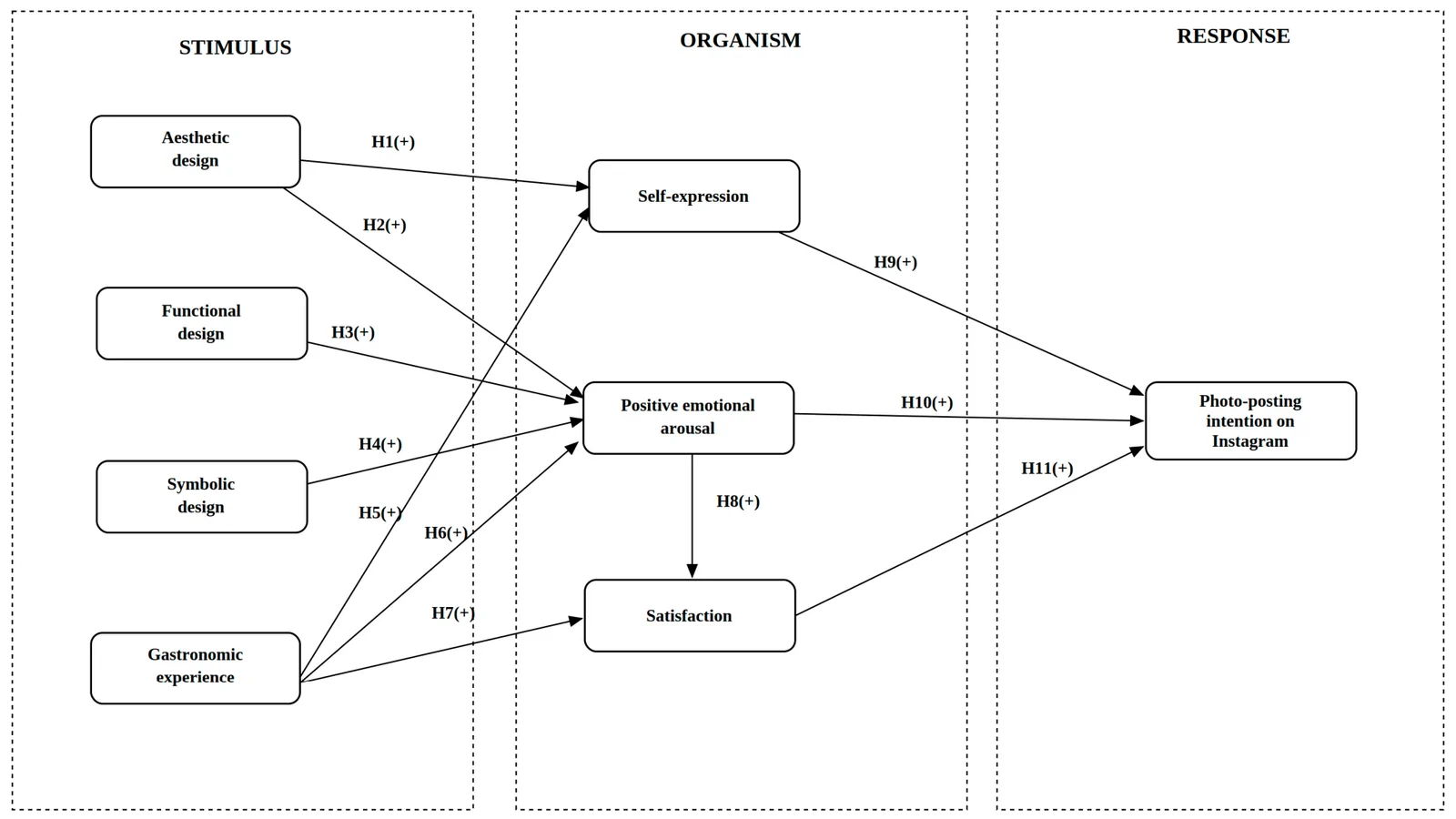
Proposed theoretical model of the antecedents of food photo-posting intention on Instagram.

**Table 1:** Measurement scales, items per construct, and source references.

Construct	Role	Items	Source
Aesthetic design	Stimulus	4	[38,39]
Functional design	Stimulus	3	[38,39]
Symbolic design	Stimulus	4	[38,39]
Gastronomic experience	Stimulus	7	[54]
Self-expression	Organism	4	[76]
Positive emotional arousal	Organism	3	[33]
Satisfaction	Organism	3	[69]
Photo-posting intention	Response	3	[39]

**Table 2:** Sociodemographic profile of the sample (n = 243).

Sociodemographic Variable	Category	Frequency	Percentage
Gender	Female	137	56.4%
	Male	103	42.4%
	Non-binary	2	0.8%
	Prefer not to say	1	0.4%
Monthly income (CLP)	Below 440,000 CLP (~USD 506)	51	21.0%
	440,001–880,000 CLP (~USD 506–1011)	37	15.2%
	880,001–1,320,000 CLP (~USD 1011–1517)	61	25.1%
	1,320,001–1,760,000 CLP (~USD 1517–2023)	39	16.0%
	1,760,001–2,200,000 CLP (~USD 2023–2529)	22	9.1%
	Above 2,200,000 CLP (~USD 2529)	33	13.6%
Restaurant visits (last 6 months)	2–3 times per month	112	46.1%
	Once per month	68	28.0%
	4–6 times per month	39	16.0%
	More than 6 times per month	24	9.9%

**Table 3:** Measurement model: factor loadings, reliability, and convergent validity (n = 243).

Construct	Indicator	Outer Loading	Cronbach’s α	CR (rho_a)	CR (rho_c)	AVE
Aesthetic design (DE)	DE1	0.914	0.960	0.961	0.971	0.894
	DE2	0.956				
	DE3	0.961				
	DE4	0.950				
Functional design (DF)	DF1	0.958	0.962	0.965	0.975	0.929
	DF2	0.967				
	DF3	0.967				
Symbolic design (DS)	DS1	0.842	0.902	0.911	0.931	0.772
	DS2	0.913				
	DS3	0.903				
	DS4	0.853				
Gastronomic experience (EG)	EG1	0.879	0.966	0.970	0.972	0.834
	EG2	0.949				
	EG3	0.960				
	EG4	0.957				
	EG5	0.932				
	EG6	0.892				
	EG7	0.813				
Positive emotional arousal (EP)	EP1	0.963	0.950	0.950	0.968	0.909
	EP2	0.954				
	EP3	0.943				
Self-expression (A)	A1	0.922	0.934	0.936	0.953	0.835
	A2	0.918				
	A3	0.905				
	A4	0.911				
Satisfaction (S)	S1	0.988	0.989	0.989	0.992	0.978
	S2	0.988				
	S3	0.990				
Photo-posting intention (I)	I1	0.966	0.961	0.962	0.975	0.928
	I2	0.974				
	I3	0.950				

Note. CR = composite reliability; AVE = average variance extracted. Source: Own elaboration.

**Table 4:** Discriminant validity: Fornell–Larcker criterion.

Construct	A	DE	DF	DS	EG	EP	I	S
Self-expression (A)	0.914							
Aesthetic design (DE)	0.308	0.945						
Functional design (DF)	0.345	0.635	0.964					
Symbolic design (DS)	0.468	0.483	0.548	0.878				
Gastronomic experience (EG)	0.416	0.626	0.660	0.489	0.913			
Positive emotional arousal (EP)	0.543	0.600	0.571	0.495	0.748	0.953		
Photo-posting intention (I)	0.717	0.458	0.459	0.476	0.549	0.605	0.963	
Satisfaction (S)	0.406	0.632	0.588	0.456	0.801	0.775	0.563	0.989

**Table 5:** Discriminant validity: Heterotrait–Monotrait ratio (HTMT).

Construct	A	DE	DF	DS	EG	EP	I	S
Self-expression (A)								
Aesthetic design (DE)	0.324							
Functional design (DF)	0.363	0.659						
Symbolic design (DS)	0.509	0.504	0.582					
Gastronomic experience (EG)	0.437	0.647	0.683	0.519				
Positive emotional arousal (EP)	0.576	0.628	0.596	0.526	0.780			
Photo-posting intention (I)	0.755	0.476	0.476	0.506	0.570	0.633		
Satisfaction (S)	0.420	0.648	0.602	0.477	0.817	0.800	0.578	

**Table 6:** Model fit indices.

Index	Saturated Model	Estimated Model
SRMR	0.040	0.072
d_ULS	0.793	2.603
d_G	0.960	1.304
Chi-square	1351.431	1393.402
NFI	0.877	0.873

**Table 7:** Coefficient of determination (R^2^).

Construct	R^2^	Adjusted R^2^	Level
Self-expression (A)	0.177	0.170	Weak
Positive emotional arousal (EP)	0.600	0.593	Moderate
Photo-posting intention (I)	0.606	0.601	Moderate–substantial
Satisfaction (S)	0.711	0.709	Substantial

**Table 8:** Structural model results and hypothesis testing.

Hypothesis	β	Sample Mean	SD	t-Statistic	*p*-Value	Decision
H1. Aesthetic design → Self-expression	0.078	0.078	0.081	0.963	0.335	Rejected
H2. Aesthetic design → Positive emotional arousal	0.174	0.172	0.059	2.941	0.003	Supported
H3. Functional design → Positive emotional arousal	0.018	0.017	0.065	0.276	0.782	Rejected
H4. Symbolic design → Positive emotional arousal	0.124	0.125	0.058	2.142	0.032	Supported
H5. Gastronomic experience → Self-expression	0.367	0.367	0.074	4.982	<0.001	Supported
H6. Gastronomic experience → Positive emotional arousal	0.567	0.565	0.064	8.862	<0.001	Supported
H7. Gastronomic experience → Satisfaction	0.501	0.501	0.086	5.844	<0.001	Supported
H8. Positive emotional arousal → Satisfaction	0.400	0.399	0.089	4.498	<0.001	Supported
H9. Self-expression → Photo-posting intention	0.556	0.554	0.053	10.580	<0.001	Supported
H10. Positive emotional arousal → Photo-posting intention	0.104	0.109	0.086	1.210	0.226	Rejected
H11. Satisfaction → Photo-posting intention	0.257	0.252	0.078	3.319	0.001	Supported

Note. β = standardized path coefficient; SD = standard deviation; bootstrapping with 5000 subsamples. Source: Own elaboration.

## Data Availability

The original contributions presented in the study are included in the article. The data are not publicly available due to privacy concerns related to participant information and ethical restrictions outlined in the research protocol. For further inquiries, please contact the corresponding author.

## Supplementary Materials

The following supporting information can be downloaded at https://www.mdpi.com/article/10.3390/nu18213624/s1. Table S1: Measurement items and sources; Table S2: Inter-item correlation matrix; Table S3: Within-construct inter-item correlations and item diagnostics; Table S4: Sensitivity analysis of structural path coefficients under alternative measurement specifications.
